# Extracellular vesicles from water kefir can interact with human neurons in vitro: a potential explanation for the role of probiotics consumption in mental health

**DOI:** 10.3934/Neuroscience.2025019

**Published:** 2025-08-21

**Authors:** Lora J. Kasselman, Saba Ahmed, Ariel De Leon, Maryann Johnson, Ankita Srivastava, Apoorva Vashisht, Heather A. Renna, Thomas Palaia, Aaron Pinkhasov, Allison B. Reiss

**Affiliations:** 1 Hackensack Meridian Jersey Shore University Medical Center, Hackensack, NJ, USA; 2 Department of Foundations of Medicine, NYU Grossman Long Island School of Medicine, Mineola, NY, 11501, USA; 3 Department of Medicine, NYU Grossman Long Island School of Medicine, Mineola, NY, 11501, USA; 4 Department of Psychiatry, NYU Grossman Long Island School of Medicine, Mineola, NY, 11501, USA

**Keywords:** mental health, depression, fermentation, culture, cognition, gut-brain axis

## Abstract

Major depressive disorder is one of the most burdensome mental health disorders. Probiotics have been shown to ameliorate depressive symptoms, though the mechanism remains unclear. This study was conducted to investigate whether extracellular vesicles (EVs) extracted from the probiotic beverage water kefir could influence gene and protein expression in human-derived neuroblastoma cells in vitro. EVs were extracted from lab-cultured water kefir and a control solution without water kefir grains by ultracentrifugation. Water kefir vesicles were imaged via electron microscopy. Neuroblastoma, microglia, and neuroblastoma-microglia co-cultures were exposed to water kefir EVs or negative control medium. Uptake of water kefir EVs was identified by microscopy. All conditions were quantified for brain derived neurotrophic factor, fractalkine, and synaptophysin RNA and protein. Data were analyzed using factorial ANOVAs with significance set at 0.05. Water kefir vesicles were taken up by neuroblastoma cells, and incubation in neuroblastoma-microglia co-culture resulted in significantly higher levels of fractalkine protein compared to media-only control (p = 0.029). To our knowledge, this is the first study to identify potential interactions between EVs derived from the probiotic beverage water kefir and human neuronal cells. Further research is needed to fully elucidate the role played by probiotic-derived EVs in human health.

## 1. Introduction

According to the World Health Organization, major depression is the most burdensome of the mental health and behavioral disorders, with about 5% of adults worldwide suffering from depression [1]. Current medical treatments for depression include anti-depressants, which are associated with numerous side effects, are stigmatizd, and expensive, all of which may lead to non-compliance, presenting a significant challenge for clinical management [2]–[4]. Furthermore, psychosocial treatments for depression can be expensive and time-consuming, are stigmatized, and rely upon individual motivation to change.

Patients with depressive disorders show decreased levels of brain derived neurotrophic factor (BDNF) in brain and serum, and successful response to traditional medical treatment correlates with its increase [5]. Depression is also associated with changes in microRNA levels, which in turn affect BDNF expression [6]–[8]. BDNF levels are also influenced by microglia and their interaction with neurons via fractalkine (CX3CR/L1) signaling [9]. Microglial damage or activation has been associated with depression and depression-like states in humans and animal models, respectively [10]. Fractalkine affects multiple protein cascades in the brain affecting mood, behavior, and cognition, including beta amyloid and tau proteins [11]–[13]. Other animal models of depression are associated with not only changes in microglia, but also alterations in neuronal synapse markers (e.g. synaptophysin), which can help identify the location and type of neurons impacted [14].

Alternative treatment of depression with non-pharmacological compounds, such as probiotics, is not the current standard of care [15]. However, studies have shown that consumption of probiotic capsules significantly decreased depressive symptoms and reduced negative thoughts associated with a sad mood [16],[17]. Additionally, oral probiotic consumption has been shown to increase BDNF levels [18]. Consumption of probiotic beverages has long been part of human culture. One example is water kefir, which is made from fermenting simple carbohydrate solutions together with water kefir grains, creating a bubbly beverage [19]. Water kefir is composed of distinct bacteria and yeast symbiotically cohabitating in translucent grains, composed primarily of a polysaccharide matrix [20],[21]. The exact microbial composition of water kefir varies depending on region of origin, preparation techniques, and the time allowed to ferment [22]. There is some evidence that the consumption of probiotics, including water kefir, can impact the composition of the gut microbiome [23]–[25].

While emerging evidence suggests that the gut microbiome is important in regulating mental health, the mechanisms are not well understood [26]. Ferrari et al. used cultured SH-SY5Y human neuroblastoma cells exposed to L-glutamate to induce inflammatory damage comparable to that found with depressive moods and demonstrated that adding a combination of probiotics than a single strain to the culture medium was superior in reducing the L-glutamate-induced inflammatory cytokine expression [27],[28]. Based on their results, they postulate that probiotic strains consumed together, as would occur in a fermented food or drink, would be a superior anti-depressant compared to a single type of probiotic in isolation. Magistrelli et al. isolated peripheral blood mononuclear cells (PBMC) from persons with Parkinson's disease and healthy controls and looked at the effect of probiotic strains on cytokine release and reactive oxygen species production by the PBMCs in vitro [29]. They found that probiotic bacteria, particularly *Lactobacillus salivarius* and *Lactobacillus acidophilus*, reduced inflammatory cytokine levels and increased anti-inflammatory cytokine levels in both Parkinson's disease and control PBMC and may, therefore, be useful in controlling neuroinflammation in the setting of Parkinson's disease.

Our knowledge of the nature of the extracellular microvesicles (EVs) produced by bacteria present in kefir is expanding as interest grows in the role of fermented products in gut health and overall health [30]. EVs isolated from a bacterial strain found in kefir can exert anti-inflammatory effects on both a human intestinal epithelial cell line and on murine models of colitis and inflammatory bowel disease [31],[32]. This reflects the ability of bacterial EVs to communicate signals to human or mouse cells. Probiotic organisms in the gut microbiome typically do not interact directly with gut primary afferent neurons, but they affect them, in part, via EVs [33]. EVs are extremely small (50–150 nm) [26], carry microRNAs, have the ability to cross the blood-brain barrier, and affect different cell types, including neurons, microglia, and astroglia [34],[35]. Microvesicle interactions with these cells in the brain may underlie the positive effects of probiotic supplementation or consumption on mental health. In fact, in an animal model of depression, probiotic EVs delivered intraperitoneally resulted in decreased depression-like behavior and increased expression of BDNF in the hippocampus [36]. Thus, our aim of this study is to determine whether EVs extracted from the probiotic drink water kefir can be taken up by human neuroblastoma cells in vitro and effect changes in BDNF, fractalkine, and synaptophysin, three proteins known to support neuronal functioning. Evidence of this nature will help fill the mechanistic gap in our understanding of how probiotics interact with the brain and subsequently contribute to mental health. Additionally, interventions with probiotics offer a low-cost method to improve mental health in the public realm.

## 2. Materials and methods

Overall, water kefir grains and a negative control (without kefir grains) were cultured, and extracellular vesicles were extracted. Vesicles from kefir and the negative control were cultured with either human neuroblastoma cells, human microglial cells, or a combination of the two. RNA and protein were then measured. Additionally, extracellular vesicles were labeled and imaged alone and after culturing with human neuroblastoma cells.

### 2.1. Water kefir maintenance

Water kefir grains (manufacturer ©Cultures for Health) were purchased online from Amazon.com in dehydrated form. They are organically grown gluten-free, GMO-free, and dairy-free. Cultures were maintained by feeding three times per week (on Mondays, Wednesdays, and Fridays) with 500 mL of deionized water, 30 g of raw sugar, and 0.08 g of calcium carbonate and allowed to sit covered at room temperature. The negative control medium contained the same amount of water, raw sugar, and calcium carbonate, but without water kefir grains, and was changed on the same schedule.

The microbial composition of water kefir is variable and may depend on several factors, such as the source of the water kefir grains, the composition of the substrate used for fermentation, and the fermentation conditions (temperature, fermentation time, etc.) [37],[38]. The water kefir grains used in this study were purchased from an online store as indicated and grown according to manufacturer's directions.

### 2.2. Isolation of EVs

Experimental procedures for this study were implemented as far as possible in accordance with the recommendations of MISEV2023 [39].

**Figure 1.**
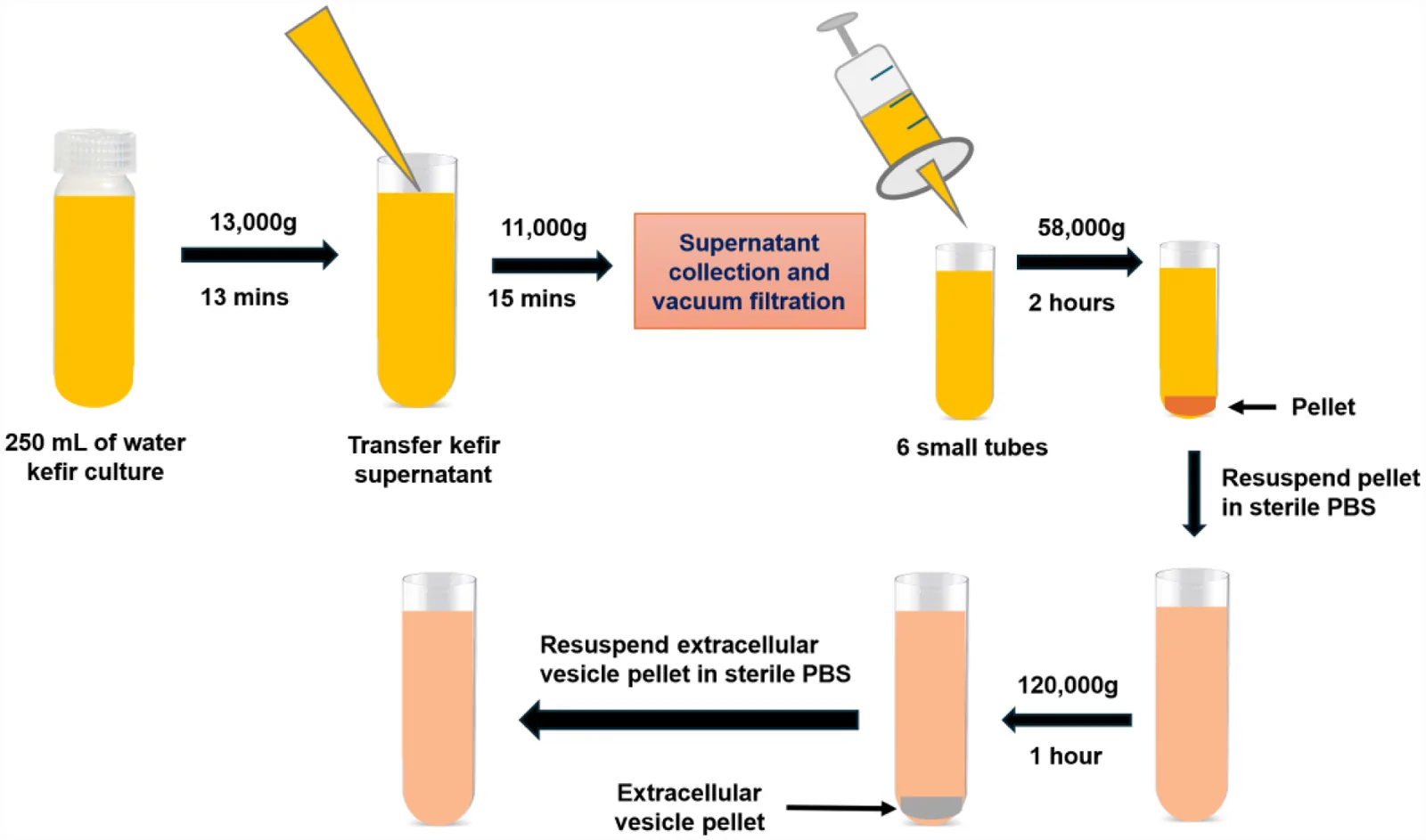
Extracellular vesicle isolation from water kefir beverage. Sequential steps of centrifugation.

A total of 250 mL of water kefir culture (excluding water kefir grains) or negative control medium were distributed among centrifuge tubes and spun for 13 minutes at 13,000 g at 22 °C with the brake off. The supernatant was collected and added to new centrifuge tubes and spun for 15 minutes at 11,000 g at 22 °C with the brake off. The supernatant was then filtered using vacuum filtration. A syringe and a syringe filter (0.2 micron) were used to transfer the contents into six smaller centrifuge tubes and spun for 2 hours at 58,000 g at 22 °C with the brake off. After completion, supernatant was aspirated, and the pellets were resuspended in sterile PBS. The resuspension was transferred into three ultracentrifuge tubes and spun with rotor SW50.2 for 1 hour at 120,000 g and 22 °C. After completion, supernatant was aspirated and the pellets were resuspended in sterile PBS (Figure 1). The resuspension protein concentration was measured using the NanoDrop One (Fisher Scientific, Waltham, MA) and was then placed in a sterile tube and concentration quantified. These samples were stored at −80 °C until needed.

### 2.3. Cell culture and experimental conditions

Human neuroblastoma, SH-SY5Y, cells (American Type Culture Collection, Manassas, VA) were cultured in DMEM-F12 media, and human microglial, HMC3, cells (American Type Culture Collection, Manassas, VA) were cultured in DMEM media. Both media were supplemented with 10% EV-free fetal calf serum (FCS), 2 mM L-glutamine, and 50 µg per mL of penicillin-streptomycin at 37 °C in a 5% CO_2_ atmosphere to a density of 500,000 cells per mL. EV-free FCS was chosen to prevent the introduction of external EVs. Cell culture media and supplementary reagents were obtained from Invitrogen (Grand Island, NY).

Then, 12-well cell culture plates were used for EV treatment of SH-SY5Y cells alone (125,000 cells/mL; n = 10), HMC3 cells alone (62,500 cells/mL; n = 7), and a co-culture of the two cell types using a hanging transmembrane (Transwell) insert (at respective single densities; Corning, Kennebunk, ME; n = 10). Each of these cell combinations were treated with either their respective media alone, the addition of 0.02 mg/mL of negative control medium, or the addition of 0.02 mg/mL water kefir EVs. The control medium was identical in composition to the kefir medium thus enabling us to distinguish the impact of bacteria/yeast from kefir from any potential effect of other components such as the sugar or water.

### 2.4. RNA isolation and QRT-PCR analysis

Following 18–24 hours of EV or negative control medium treatment, whole cell lysates were collected with intracellular RNA isolated using Trizol Reagent (Fisher Scientific, Waltham, MA). RNA concentrations were quantified with the NanoDrop One (Fisher Scientific, Waltham, MA) and standardized to a concentration of 1 µg/mL. RNA was then reverse transcribed to produce cDNA using the Mastercycler Nexus Gradient (Eppendorf, Hamburg, Germany). qPCR was performed with specific primers (Table 1) using a Roche Lightcycler 480 system to quantify the levels of gene expression for CX3CL1, BDNF, and Synaptophysin. Each reaction was executed in triplicate and levels of gene expression were normalized to the housekeeping gene glyceraldehyde 3-phosphate dehydrogenase (GAPDH). A melting-curve analysis was performed to assess the specificity of the amplified PCR products.

**Table 1.** Primer sequences for PCR analysis of mRNA expression.

**Gene**	**Forward sequence**	**Reverse Sequence**
BDNF	AGCTATCCAGAGCATCTTCCA	ACCTGGTGGAACTTTATGAAACC
Synaptophysin	AAGGGTGTGGCTTGGAACAT	GTCCCAAGTCGTAGCCAGAG
CX3CL1	CTCCGATATCTCTGTCGTGGC	ATGTTGCATITCGTCACACCG

### 2.5. Western blotting

Protein was collected from whole cell lysates that were harvested following 24 hours of treatment using radioimmunoprecipitation assay (RIPA) lysis buffer (98% PBS, 1% Igepal, 0.5% sodium deoxycholate, 0.1% sodium dodecyl sulfate, supplemented with 10 µL per mL of protease inhibitor cocktail, Sigma). Protein content was measured in triplicate using the BCA Protein Assay Kit by absorption at 562 nanometers (Pierce Biotechnology Inc., Rockford, IL, USA). Whole-cell lysate protein extracts were separated and analyzed by 12% SDS-polyacrylamide gel electrophoresis (SDS-PAGE). A total of 6.25 µg of each sample was loaded per gel lane and transferred to PVDF membranes. The blots were subsequently blocked with 5% milk in tris-buffered saline with Tween-20 (TBST) for 1 h at room temperature and incubated in primary antibodies at 4 °C overnight. For immunoblot analysis, proteins were probed with primary antibodies diluted in 5% milk in TBST against synaptophysin (Abcam catalog number ab32127, rabbit monoclonal, 1:20,000 dilution) and CX3CL1 (NBP1-49539, rabbit polyclonal, 1:1000 dilution). β-actin (Cell Signaling Technologies, CST3700, mouse monoclonal) diluted at 1:1000 in 5% milk in TBST was used as the control. Bound antibodies were visualized with a 1:2000 dilution of their respective horseradish peroxidase-conjugated secondary antibodies prepared in 1% milk in TBST. The immunoreactive protein was detected using ECL western blotting detection reagents (Thermo Scientific™ SuperSignal™ West Pico PLUS Chemiluminescent Substrate and the Bio-Rad ChemiDoc Touch Imaging System). Protein expression was normalized to the expression of β-actin and quantified via densitometry with ImageJ software (NIH, Maryland, USA).

### 2.6. Fluorescent dye staining

#### 2.6.1. EV staining

PKH26 lipophilic red fluorescent dye (Mini26-KT, Sigma) was prepared at a concentration of 0.2 µM to a final volume of 1.2 mL in Diluent C (a salt-free isotonic solution). To begin the labeling reaction, the dye was added in a 1:1 ratio with water kefir EVs suspended in sterile PBS. The labeling reaction was stopped by adding 2% BSA solution in sterile PBS in a 1:1 ratio for a final concentration of 1% BSA. The samples were centrifuged twice at 120,000 g for 2 hours to remove unbound dye. After centrifugation, the samples were resuspended in the original amount of growth media (DMEM F-12 completed with EV-depleted FCS).

#### 2.6.2. SHSY-5Y staining

Neuroblastoma cell staining: Human neuroblastoma (SH-SY5Y) cells were incubated with 1 mL of the fluorescently-labeled EVs for 1.5 hours at 37 °C and then washed with sterile PBS for 5 minutes, four times. Subsequently, the cells were fixed in 10% buffered formalin for 20 minutes and then washed with sterile PBS for 5 minutes, three times. Slides were each blocked with 1 mL of 1% BSA, 10% goat serum & 0.1% Tween-20 in PBS for 1 hour at room temperature and then rinsed with 0.025% TritonX in PBS three times, for 5 minutes each. Slides were then incubated in a 1:1000 dilution of FITC-labeled anti-α tubulin (Abcam, catalog number ab7291) in 1% BSA, 10% goat serum and 0.1% Tween-20 in PBS overnight at 4 °C. After incubation the slides were rinsed for 5 minutes with 0.025% TritonX in PBS, 3 times. DAPI Vectashield (Vector Laboratories, Burlingame, CA) was used in order to prepare a 1:1 concentration of DAPI solution in PBS, which was applied to the fixed labeled neuroblastoma cells, covered using a coverslip, and stored at 4 °C until imaged.

### 2.7. Microscopy

#### 2.7.1. Confocal microscopy

EVs labeled with PKH26 red fluorescent dye were prepared as described above and observed using the Nikon Eclipse Ti confocal microscope (Nikon, Melville, NY USA). Images were captured using the NIS Elements application with a scale bar of 20 micrometers. Uptake of EVs in cells was noted as proof-of-concept via confocal fluorescence overlap.

#### 2.7.2. Electron microscopy

EV were isolated by multiple centrifugations as described above and in Figure 1. The EV pellet was washed with PBS and fixed in 4% glutaraldehyde/0.1 M Na cacodylate buffer, pH 7.4 for 1 hour. A drop of the re-suspended extracellular vesicle suspension was then placed on parafilm and a formvar coated 300 mesh copper transmission electron microscopy grid, floating for 10 minutes, to enable the EVs to bind to the formvar. The grids were then floated on 3 separated PBS drops for washing, placed on a drop of 1% aqueous Uranyl Acetate for 10 minutes, and washed again with PBS before air drying overnight in the dark. Stained grids were examined on a Zeiss EM 900 transmission electron microscope retrofitted with an SIA L3C digital camera (SIA, Duluth, GA).

### 2.8. Data analysis

Statistical analysis was performed using Graphpad Prism, version 6 (GraphPad Software, San Diego, CA). All data were analyzed by factorial ANOVA. Probability values less than 0.05 were regarded as significant. Data are presented as mean ± standard deviation unless otherwise specified. QRT-PCR data was analyzed using dCt values (GAPDH Ct – gene of interest Ct), and then the 2ΔΔCt method was used to calculate fold change over control. PCR data are presented as fold change with corresponding 95% confidence intervals. Samples were not used for statistical analysis if RNA quality was low or if protein levels were too low for Western blot quantification.

## 3. Results

### 3.1. Microscopy

**Figure 2.**
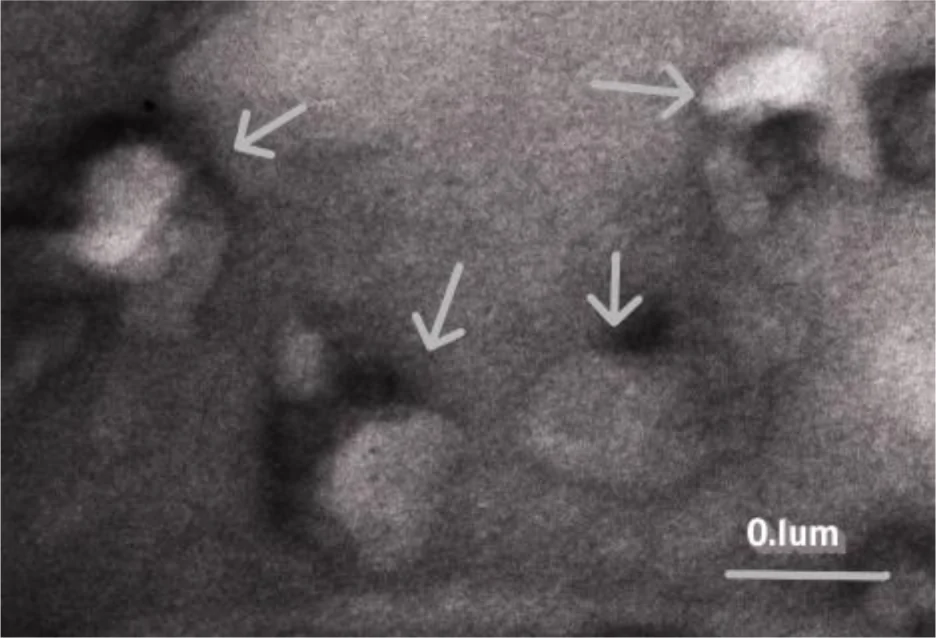
Electron microscopy image of extracellular vesicles extracted from the probiotic beverage water kefir. Grey arrows highlight extracellular vesicles. Scale bar = 0.1 micrometers.

**Figure 3.**
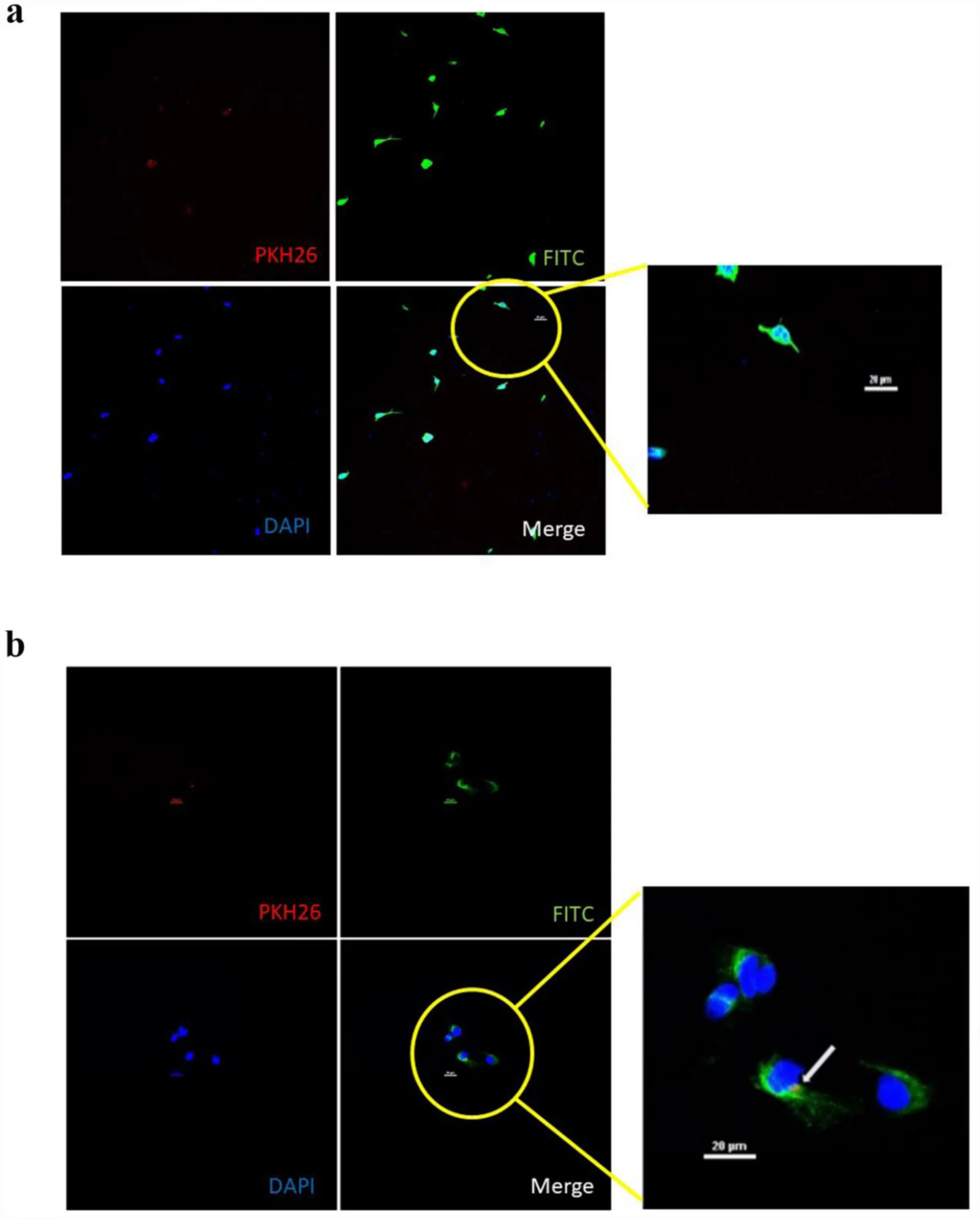
a. Confocal microscopy image of dyed SH-SY5Y neuroblastoma cells (green, FITC) with DAPI-stained nuclei (blue) and dyed extracellular vesicles extracted from a control solution (red, PKH26). Scale bar = 20 micrometers. b. Confocal microscopy image of dyed SH-SY5Y neuroblastoma cells (green, FITC) with DAPI-stained nuclei (blue) and dyed extracellular vesicles extracted from the probiotic beverage water kefir (red, PKH26). The white arrow highlights an extracellular vesicle co-localized inside a neuroblastoma cell. Scale bar = 20 micrometers.

Electron microscopy shows the presence of EVs extracted from water kefir supernatant (Figure 2). Electron microscopy is the definitive gold standard method for EV identification as it gives detailed information on the size, shape, and morphology of EVs [40],[41]. Uptake of dyed water kefir EVs in fluorescence-labeled SH-SY5Y cells was noted via merged confocal microscopy showing the overlay of fluorescently dyed neuroblastoma cells and negative control medium and water kefir EVs, with the latter indicating colocalization (Figures 3a,b, respectively).

### 3.2. Gene expression

**Figure 4.**
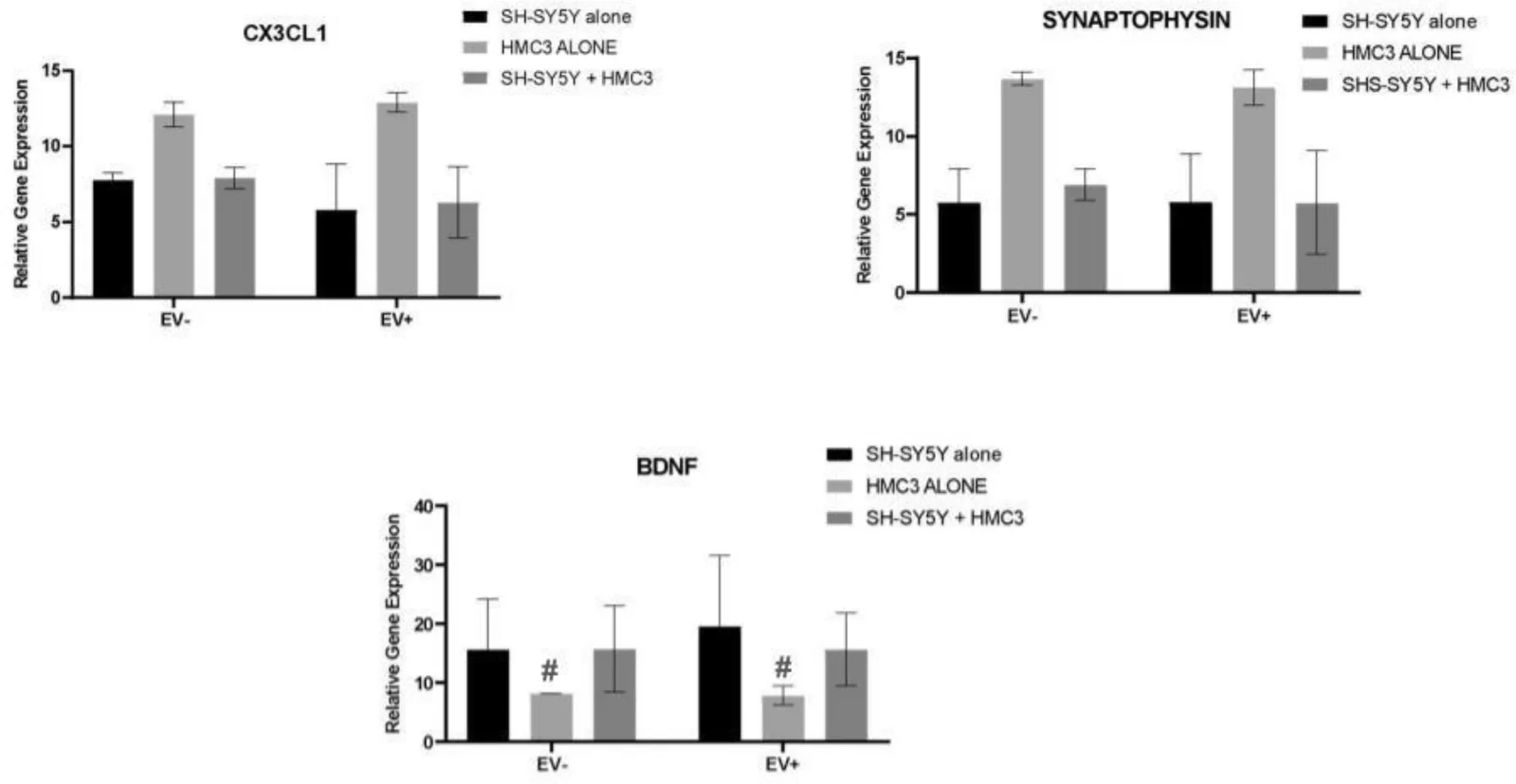
Quantitative real-time PCR analysis of CX3CL1, Synaptophysin, and BDNF in SH-SY5Y cells, HMC3 cells, and SH-SY5Y+HMC3 co-culture with (EV+; n range: 2–10) or without (EV-; n range: 2–5) EVs. GAPDH was used as internal control. Data was verified by three independent experiments. #p < 0.10 (indicating a trend, not significance) vs. SH-SY5Y cells and SH-SY5Y+HMC3 co-culture.

There was no significant interaction between cell type and extracellular vesicle treatment in BDNF expression [F(2,29) = 0.1427, p = 0.8676]. There was a trend for HMC3 cells to have lower expression of BDNF compared to SH-SY5Y cells and HMC3+SH-SY5Y co-culture [F(2,29) = 3.221, p = 0.0545; Figure 4]. There was no significant difference in BDNF expression for EV treatment [F(1,29) = 0.4728, p = 0.4971]. There was no significant interaction between cell type and EV treatment in CX3CL1 expression [F(2,18) = 0.567, p = 0.5769]. HMC3 cells had significantly higher expression of CX3CL1 compared to SH-SY5Y cells and HMC3+SH-SY5Y co-culture [F(2,18) = 10.34, p = 0.0010; Figure 4]. There was no significant difference in CX3CL1 expression for EV treatment [F(1,18) = 0.7990, p = 0.3831]. There was no significant interaction between cell type and EV treatment in synaptophysin expression [F(2,29) = 0.164, p = 0.8489]. HMC3 cells had a significantly higher expression of synaptophysin compared to SH-SY5Y cells and HMC3+SH-SY5Y co-culture [F(2,29) = 17.63, p < 0.0001; Figure 4]. There was no significant difference in CX3CL1 expression for EV treatment [F(1,29) = 0.3070, p = 0.5836].

### 3.3. Protein levels

**Figure 5.**
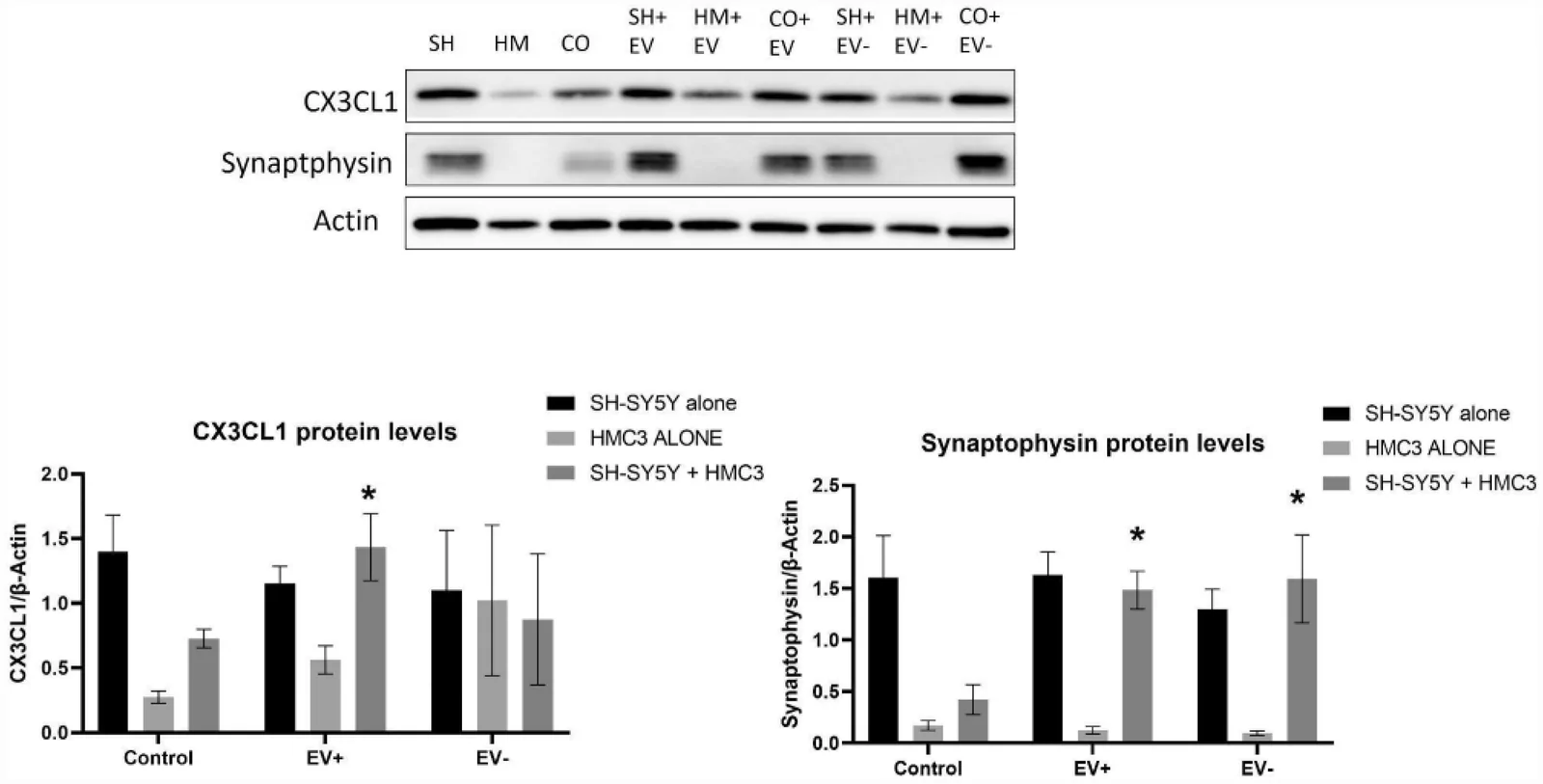
Western blot analysis of CX3CL1 and synaptophysin in SH-SY5Y cells, HMC3 cells, and SH-SY5Y+HMC3 co-culture with (EV+; n = 3) or without (EV-; n = 3) EVs. β-Actin was used as an internal control. Data was verified by three independent experiments; *p < 0.05 versus control. SH represents SH-SY5Y cells, HM represents HMC3 cells, CO represents SH-SY5Y+HMC3 co-culture, SH+EV+ represents SH-SY5Y cells with EVs, HM+EV+ represents HMC3 cells with EVs, CO+EV+ represents SH-SY5Y+HMC3 co-culture with EVs, SH+EV- represents SH-SY5Y cells without EVs, HM+EV- represents HMC3 cells without EVs, and CO+EV- represents SH-SY5Y+HMC3 co-culture without EVs.

There were no results for BDNF Western blot due to technical difficulties and low EV concentrations. There was a significant interaction between cell type and EV treatment in CX3CL1 protein levels [F(4,18) = 3.466, p = 0.0288; Figure 5]. HMC3+SH-SY5Y cells co-cultured with EVs had significantly higher CX3CL1 protein levels compared to SH-SY5Y cells and HMC3 cells alone in HMC3+SH-SY5Y co-culture [F(2,18) = 7.554, p = 0.0042; Figure 5]. There was a significant interaction between cell type and EV treatment in synaptophysin protein levels [F(4,18) = 9.773, p = 0.0002; Figure 5]. SH-SY5Y cells and HMC3+SH-SY5Y co-culture had significantly higher synaptophysin protein levels compared to HMC3 cells in both the water kefir negative control medium condition and water kefir EVs [F(2,18) = 84.66, p < 0.0001] (Figure 5).

## 4. Discussion

In summary, this study provides preliminary evidence of the presence of EVs in publicly available cultured water kefir grains, and the results suggest that human neuronal cells in vitro can uptake EVs isolated from cultured water kefir grains. The study also revealed increased levels of fractalkine protein in neuroblastoma-microglia co-cultures exposed to water kefir EVs and identified increased synaptophysin protein levels in neuroblastoma-microglia co-cultures exposed to both the water kefir negative control medium condition and water kefir EVs. To our knowledge, we are the first to identify potential interactions between EVs derived from the probiotic beverage water kefir and human neuroblastoma cells, though these results must be confirmed by future studies.

Growing evidence suggests that probiotics are vital to many aspects of human health, including metabolic function, body size, and cognitive and mental health, among others, via interaction with the gut microbiome [42]–[46]. The mechanisms of this relationship are not clearly understood, but published literature suggests several, including, but not limited to, modulation of gut immunity, short-chain fatty acids, and alterations in neurotransmitter levels [47],[48]. Another mechanism that has recently gained traction is the interaction between bacterial-secreted EVs and host cells [49]. In fact, EVs from other liquids, e.g. milk, have been shown to cross the blood brain barrier and impact neuronal function and behavior in animal models, including the amelioration of depression-like symptoms [50],[51].

Although the exact mechanism is not fully understood, there is much supporting evidence for the role of probiotics consumption on cognition and mental health. In community dwelling adults, probiotics consumption increased serum BDNF levels and improved mental flexibility compared to placebo [24]. A meta-analysis demonstrated that probiotics are associated with a significant reduction in depression [52], though similar results were not seen for anxiety in a different clinical trial [53]. In participants with coronary artery disease, supplementation with probiotics and the addition of a prebiotic resulted in improved depression and anxiety scores, along with improved inflammatory profiles [54]. A clinical trial from Elahinejad et al. revealed that in persons with major depressive disorder, probiotic supplementation added on to fluoxetine treatment for 8 weeks reduced the severity of depression compared to those on fluoxetine given a placebo [55]. In another study, participants with chronic stress who consumed a probiotic capsule for 4 weeks showed decreased inflammatory markers, including soluble fractalkine [56]. This is contrary to our findings with increased fractalkine, though we used a whole probiotic product and not an isolated probiotic strain, which may account for the difference and is a better representation for real-world consumption. Our results do show concordance with studies of bidirectional communication between neurons and microglia, in which fractalkine has been shown to play a major role in neuronal plasticity and neuroinflammation [57]. In fact, fractalkine-deficient mice exhibit a depressive-like phenotype [58].

In addition to its effect on mental health, there is growing recognition that the microbiota has a role in modulating a number of pathological states in humans. For example, the production of the short chain fatty acid butyrate via fermentation of dietary fiber by gut microbiota may improve insulin resistance and support healthy body weight [59],[60]. In the field of cancer, probiotics can alter microRNAs that affect cancer cell growth and the immune response to tumors in cancers originating in the colon and breast [61]–[63].

While evidence from this study adds to the growing literature regarding the possible mechanism(s) behind the role of probiotics in human health, there are several limitations to this study. The data used in this study are in vitro, using human cell lines, and thus need to be replicated in human neurons and microglia, or in clinical trials using water kefir as the intervention and measuring psycho-cognitive traits. However, human neuroblastoma cells have been used in other studies to test mechanistic actions of anti-depressants [64],[65]. Additionally, the concentration of EVs extracted from water kefir was extremely low, restricting the concentration to one dose, limiting the possibility of showing a dose-dependent response, and limiting more in-depth characterization of EV size distribution and content. We also encountered technical difficulties with the BDNF antibody, resulting in the inability to quantify BDNF protein, which was initially the strongest mechanistic hypothesis. The results revealed discrepancies between RNA and protein expression levels, which may indicate that there were post-transcriptional or post-translational changes not accounted for in this study [66]. Additionally, some of our data contains relatively wide error bars which may be due to a low sample size, highlighting the need for additional studies. While we extracted EVs from whole water kefir cultures and did not culture the water kefir to identify exact organisms, we could have isolated one water kefir-derived strain in order to elucidate the effects of one specific probiotic on human cells in vitro. However, we decided to use the whole extract since there may be synergistic effects of all probiotic strains in the water kefir, thus potentially resulting in a missed opportunity to find relevant results. Moreover, though isolation of specific probiotic strains would provide specific interactions between that strain and human cells in vitro, we wanted to keep the water kefir methods as close to real world consumption as possible, meaning that people would likely be drinking the whole water kefir beverage, not an isolated extract of water kefir derived EVs.

The microbial components of water kefir are generally a combination of yeast and bacteria, with *Saccharomyces cerevisiae*, *Lactobacillus* species, and *Acetobacter* species predominating [22],[67],[68]. Of these, the Lactobacilli family in particular is known to affect neurons and neurotransmitters and has been shown to improve depressed mood [69],[70].

Further in vitro and clinical research is needed to fully elucidate the role that probiotic-derived EVs play in human health. In particular, more research in humans would be essential for documentation of the role of probiotics in mental health.

## 5. Conclusions

Pharmacologic treatment of depression is difficult and carries many side effects and risks, especially over the long-term. The influence of the gut-brain axis on mood can bring new opportunities to manage mental health with fewer adverse consequences and, as key modes of intercellular communication over distance, extracellular vesicles are well-suited to convey cargo from the gastrointestinal system through the blood brain barrier to the central nervous system. Our results indicate that water kefir, a fermented probiotic drink, generates EVs that can penetrate the neuronal cell membrane and enter the neuron. These vesicles also influence protein expression. Future studies are planned using labeled water kefir EVs in a mouse model to confirm that the vesicles can reach the brain in vivo, and these experiments will enable us to assess behavioral and mood changes in a pre-clinical system. We are also planning a study involving the intestine, since, in order for the EVs in food products to reach the brain, they must first be absorbed by the intestinal epithelium, enter the bloodstream, and bind to transport proteins to cross the blood-brain barrier. Exploring the path of EVs from ingestion to the brain opens up possibilities to prevent and/or control psychiatric and neurological diseases.

## Use of AI tools declaration

The authors declare they have not used Artificial Intelligence (AI) tools in the creation of this article.
